# Clinical Application of the Hanover Classification for Iatrogenic Bile Duct Lesions

**DOI:** 10.1155/2011/612384

**Published:** 2012-01-05

**Authors:** Hüseyin Bektas, Moritz Kleine, Azad Tamac, Jürgen Klempnauer, Harald Schrem

**Affiliations:** Klinik für Allgemein, Viszeral- und Transplantationschirurgie, Medizinische Hochschule Hannover, Carl-Neuberg-Stra*β*e 1, 30625 Hanover, Germany

## Abstract

*Background*. There is only limited evidence available to justify generalized clinical classification and treatment recommendations for iatrogenic bile duct lesions. *Methods*. Data of 93 patients with iatrogenic bile duct lesions was evaluated retrospectively to analyse the variety of encountered lesions with the Hanover classification and its impact on surgical treatment and outcomes. *Results*. Bile duct lesions combined with vascular lesions were observed in 20 patients (21.5%). 18 of these patients were treated with additional partial hepatectomy while the majority were treated by hepaticojejunostomy alone (*n* = 54). Concomitant injury to the right hepatic artery resulted in additional right anatomical hemihepatectomy in 10 of 18 cases. 8 of 12 cases with type A lesions were treated with drainage alone or direct suture of the bile leak while 2 patients with a C2 lesion required a Whipple's procedure. Observed congruence between originally proposed lesion-type-specific treatment and actually performed treatment was 66–100% dependent on the category of lesion type. Hospital mortality was 3.2% (*n* = 3). *Conclusions*. The Hannover classification may be helpful to standardize the systematic description of iatrogenic bile duct lesions in order to establish evidence-based and lesion-type-specific treatment recommendations.

## 1. Introduction

Intraoperative injury of the ductus hepatocholedochus (DHC) or hepatic duct is one of the most severe complications in gallbladder surgery. The literature reports an incidence rate of 0.5–0.8% after laparoscopic cholecystectomy and an incidence rate of 0.2–0.3% after open surgical cholecystectomy [2–7]. These lesions are typically demanding for the surgeon as the pattern of injury may be complicated, for example, by concomitant vascular injuries. The treatment of such lesions is associated with a high rate of complications [1, 8]. The choice of surgical reconstruction and the timing of surgical repair are decisive for the long-term course [5]. Numerous surgical and interventional treatment modalities that are available require close interdisciplinary cooperation of gastroenterologists, radiologists, and surgeons [3, 5, 9–13].

We performed a retrospective study to demonstrate the variety of injury patterns and the subsequent therapeutic concepts for the treatment of iatrogenic bile duct lesions and their outcome. For this purpose we used the Hanover classification for iatrogenic bile duct lesions including concomitant vascular lesions [1] and reevaluated its clinical application and significance in a larger series of cases.

## 2. Patients and Methods

Data of 93 patients who were treated for iatrogenic bile duct lesions at our institution were analysed retrospectively by chart review. For the follow-up survey a questionnaire was sent to patients and general practitioners (GPs). The questionnaire for the GPs included questions on the clinical condition of the patient and cholangitis-specific laboratory parameters during follow-up. Cholangitis was defined as a number of symptoms including fever, chills, increased infection parameters, and cholestasis values.

**Figure 1:**
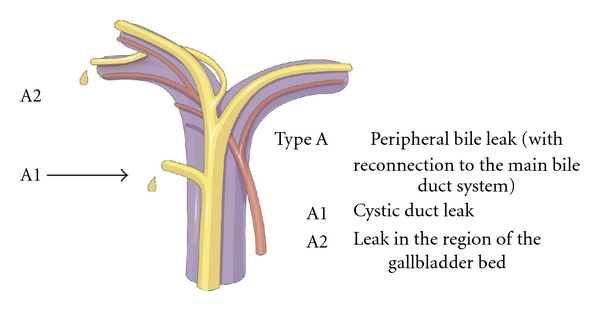
Shown is an illustration of an iatrogenic bile duct lesion which is characterized by peripheral bile leakage with connection to the main bile duct system. According to the Hanover Classification and as described and shown previously [1], such a lesion would be labelled as a type A lesion (permission to use this figure has been obtained from the publisher).

**Figure 2:**
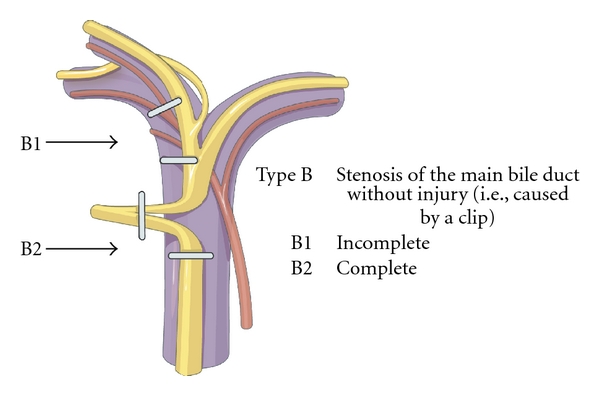
Shown is an illustration of an iatrogenic bile duct lesion which is characterized by stenosis of the common bile duct (ductus hepatocholedochus, DHC) which may be caused by a clip. According to the Hanover Classification and as described and shown previously [1], such a lesion would be labelled as a type B lesion (permission to use this figure has been obtained from the publisher).

**Figure 3:**
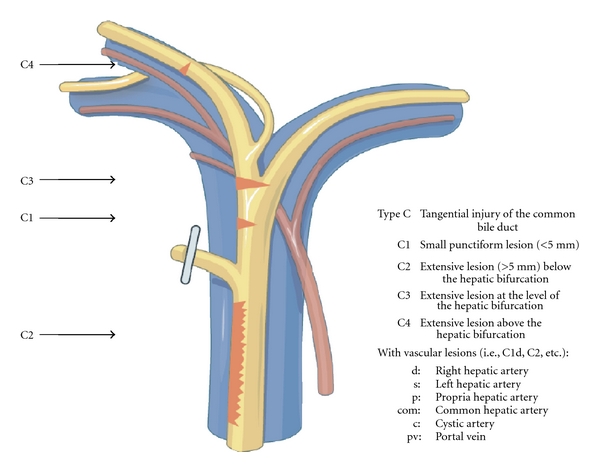
Shown is an illustration of an iatrogenic bile duct lesion which is characterized by tangential injury of the common bile duct (ductus hepatocholedochus, DHC) with or without additional vascular injury. According to the Hanover Classification and as described and shown previously [1], such a lesion would be labelled as a type C lesion (permission to use this figure has been obtained from the publisher).

**Figure 4:**
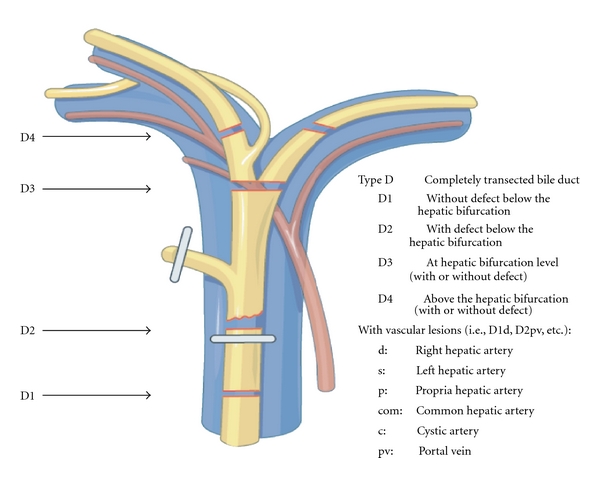
Shown is an illustration of an iatrogenic bile duct lesion which is characterized by complete transsection of the common bile duct (ductus hepatocholedochus, DHC) with or without additional vascular injury. According to the Hanover Classification and as described and shown previously [1], such a lesion would be labelled as a type D lesion (permission to use this figure has been obtained from the publisher).

**Figure 5:**
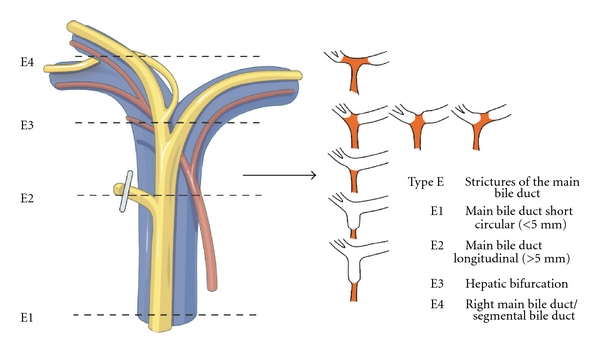
Shown is an illustration of an iatrogenic bile duct lesion which is characterized by strictures of the common bile duct (ductus hepatocholedochus, DHC). According to the Hanover Classification and as described and shown previously [1] such a lesion would be labelled as a type E lesion (permission to use this figure has been obtained from the publisher).

Patients were categorised using a new classification (Hanover classification), for which we were able to demonstrate clear advantages over the published conventional classification systems [1]. The new classification covers lesions localised above the bifurcation of the common bile duct in more detail, thus categorising patients, who were otherwise not included in any other published classification system so far. The Hanover Classification was used as described before [1] (see also Figures 1–5).

The classification of bile duct lesions, their treatment, and outcome were analysed in order to demonstrate the variety of encountered injury patterns and subsequent therapeutic concepts and their outcome in a larger series and in order to evaluate the clinical value of the Hanover Classification.

## 3. Results

The gender distribution among the 93 patients was 67 females versus 26 males. The mean age was 61 years (14–79 years.); the mean follow-up period was 53 months (2–172 months).

In 3 patients the lesions occurred in our clinic; 90 patients were referred from other hospitals after an iatrogenic bile duct lesion had been diagnosed. Iatrogenic bile duct lesions occurred after laparoscopic cholecystectomy (*n* = 76), after open cholecystectomy (*n* = 12), and after ERCP (*n* = 5). Indications for cholecystectomy were symptomatic cholecystolithiasis (*n* = 60), chronic cholecystitis (*n* = 12), acute cholecystitis (*n* = 13), contracted gallbladder (*n* = 4), gallbladder empyema (*n* = 2), and in two cases bile duct perforation during an ERCP without concomitant cholecystitis.

In 38 cases the lesion was noted immediately during the primary intervention due to bile leakage. In 23 of these 38 cases the operating surgeon mentioned possible explanations for the lesions in his operative reports: misinterpretations of the anatomy (13 cases), difficult anatomical situations (4 cases), and bile duct anatomical variance (6 cases).

Intraoperative cholangiography (IOC) was performed in 24 cases. However, in 13 cases the lesion remained unnoticed despite the IOC. According to the operative reports which were available for this study, the cystic artery was positively identified prior to transsection in 54 cases while in 8 cases the surgeon could not positively identify the cystic artery. In 59 cases the cystic duct was also positively identified; however, 11 surgeons reported that positive identification had not been possible prior to transsection (see Table 1).

**Figure 6:**
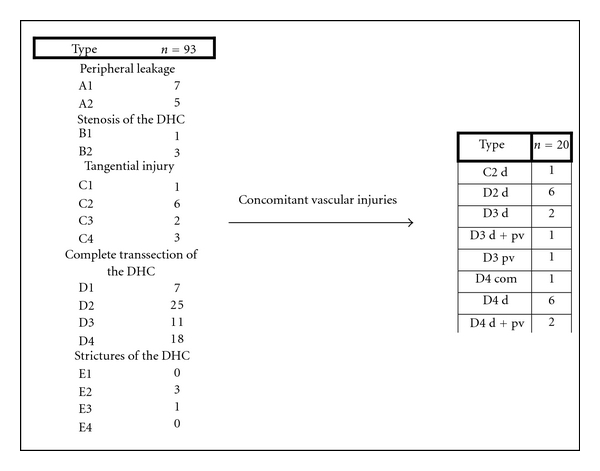
Categorisation of patients with bile duct lesions and concomitant vascular injuries according to the Hanover Classification (d = A. hep. dex; s: a. hep. sin; p: a. hep. prop.; c: a. cystica; pv: portal vein; com: a. hepatica communis; DHC: common bile duct, ductus hepatocholedochus).

Most lesions were categorised as type D lesions according to the Hanover Classification (*n* = 60). In 12 patients a tangential type C lesion was apparent (see Figure 6).

In 20 cases bile duct lesions were associated with additional vascular injuries. Arterial injury was found in 19 cases and injury of the portal vein in 4 cases. In 3 of 4 cases with portal vein injury additional injury of the right hepatic artery was found. In one case the tangential injury of the DHC was associated with a vascular lesion of the right hepatic artery (C2d lesion according to the Hannover Classification). In 6 cases with complete transsection of the DHC below, the bifurcation concomitant vascular injuries to the right hepatic artery were found (D2d lesion). In 4 cases with complete transsection at the level of the hepatic duct bifurcation additional injuries to either the right hepatic artery (D3d, *n* = 2) or the portal vein (D3pv, *n* = 1) or to both the portal vein and the right hepatic artery (D3d + pv, *n* = 1) were found. Complete transsection of the DHC above the bifurcation combined with additional vascular injuries was evident in 8 cases (D4d, *n* = 6; D4d + pv, *n* = 2) (see also Figure 6 and Figures 1, 2, 3, 4, 5). 18 of the 20 cases with additional vascular injuries were treated by surgery including liver resection.

Concomitant injury to the right hepatic artery (C2d lesion *n* = 1; D2d lesion *n* = 6; D3d lesion *n* = 2; D4d lesion *n* = 6; D3d + pv lesion *n* = 1, D4d + pv lesion *n* = 2) resulted in additional right hemihepatectomy in 10 of 18 cases. 8 of 12 cases with type A lesions were treated with drainage alone or direct suture of the bile leak while 2 patients with a C2 lesion required a Whipple's procedure (see Table 3).

Only 8 of 93 patients primarily underwent endoscopic therapy only. However, during the later course surgery was indicated in all of these cases due to development of strictures, cholestasis, or recurrent cholangitis.  Table 2 shows data of the primary and the definitive interventions performed in our clinic in order to treat the iatrogenic lesion.

In our hospital first-line operative therapy of iatrogenic bile duct lesions was undertaken in 72 patients 0–11 days after the initial injury. Further 21 patients were referred to our hospital for surgical therapy with long-term complications like anastomotic stricture or bile duct stenosis after iatrogenic bile duct lesions (range: 1–15 years after bile duct injury) (for details see Table 2). Among all 93 patients treated in our hospital, hepaticojejunostomy was performed in 53 patients, one patient was treated with arterial reconstruction by primary suture plus hepaticojejunostomy, 14 patients received a hemihepatectomy plus hepaticojejunostomy (one of these procedures was already done prior to referral to us for biliary stricture), nine patients were treated with re-hepaticojejunostomy, four patients were treated by liver resection alone (right hemihepatectomy two cases, segmental liver resection two cases), two patients were operated with a Whipple's procedure due to intrapancreatic injury to the bile duct, one patient was treated by drainage only, three patients were treated with exploratory laparotomy and adhesiolysis, five patients were treated by direct suture of the bile duct lesion after exploratory laparotomy, and in two patients the simple removal of a clip was sufficient after surgical exploration.

Early complications during the first hospitalisation in our institution requiring urgent operative revision occurred in 19 patients (20.5%) (Table 2). One patient needed urgent operative revision due to postoperative thrombosis of the hepatic artery and the portal vein followed by urgent liver transplantation. Other early complications leading to urgent operative revisions included secondary haemorrhage, bile leakage, anastomotic insufficiency of the hepaticojejunostomy, peritonitis, and duodenal perforation (see Table 2). The mean duration of hospitalisation was 16 days (3–116 days) with a mean stay in the ICU of 2 days (0–116 days).

12 patients required additional reconstructive surgery in our clinic during long-term follow-up, 4 patients needed re-hepaticojejunostomy, and 7 patients had to undergo herniotomy due to an incisional hernia. Liver transplantation was performed in one other case due to chronic secondary sclerosing cholangitis. Further, closure of a tracheostoma was performed in two cases, partial resection of the liver due to recurrent cholangitis following injury of the right hepatic artery and adhesiolysis due to adhesive ileus was necessary in one patient each. Long-term follow-up data of 63 patients was available.

## 4. Discussion

Diagnosis and therapy of iatrogenic bile duct lesions are a challenge for the surgeon [5, 8, 9, 11, 13, 14]. Less than 50% of these lesions are detected and treated adequately during cholecystectomy. The majority of lesions are noticed at a later stage during hospitalisation or later due to their imminent sequelae which may become apparent sometimes months after the cholecystectomy has been performed [6, 9, 15–17]. In 41% (*n* = 38) of our cases lesions were detected while the cholecystectomy was being performed; other centres report similar numbers [15, 16].

Our series with a broad variety of different injury patterns of the central bile ducts with and without concomitant vascular involvement clearly demonstrates a great variance in the scope, extent, and invasiveness of surgical interventions for the treatment of iatrogenic bile duct lesions and highlights the amount of complexity in specific care. Taken together it appears obvious that the complexity of heterogeneity in bile duct lesions and their therapy requires a systematic approach for which we have developed the Hanover Classification in order to help the clinician to develop a rationale for decision making in these patients [1] (see Table 3).

We attempted to investigate to what degree the primary therapy of iatrogenic bile duct lesions was actually in line with the initial procedures that we have proposed for specific types of bile duct lesions according to the Hanover Classification as outlined in our previous publication (see Table 3). It is important to note in this context that the vast majority of primary interventions were carried out prior to referral to us. Interestingly, for type A, type B, type C, type D and type E lesions the previously proposed therapy and the actual primary intervention were identical in 62%, 75%, 90%, 72%, and 100% of cases, respectively (see Table 3). We assume that the majority of surgeons in our area follow the treatment proposals for different iatrogenic bile duct lesions as outlined in our proposed Hanover Classification. Still many patients were referred to us with clinical problems after an initial surgical attempt to treat the bile duct lesion locally first. We believe therefore that the treatment of complicated iatrogenic biliary lesions frequently requires specialist surgical experience in hepatobiliary surgery. We assume that a large but unknown proportion of cases with iatrogenic bile duct lesions were treated successfully locally and were therefore not referred to us or any other centre. We believe that this assumption warrants further investigation.

The frequency of iatrogenic bile duct lesions with additional vascular lesions is reported to be 11–32% [18, 19]. In our collective concomitant vascular injuries were evident in 20 of 93 patients (21.5%). Apart from four cases with portal vein injury, most vascular injuries affected the right hepatic artery. Detection and adequate treatment of these concomitant injuries are essential for the long-term course as the main blood supply to the bile duct system is from the right hepatic artery. Alves et al. reported that they observed no significant differences in the long-term course of patients with postoperative biliary complications, either with or without arterial lesions [7]. In contrast, Schmidt et al. showed that injury of the right hepatic artery increases the risk for the development of biliary complications [8]. In our view injury of the hepatic artery has to be seen as potentially life threatening. It was found that in all three patients who died during hospitalisation a concomitant injury to the common hepatic artery had been diagnosed. We advocate therefore a preoperative angio-CT of the liver. This ensures that the planned operation can be adapted as necessary if there is evidence of a concomitant vascular injury. Intraoperative identification of the hilar structures is frequently seriously complicated by previous infection and previous surgery. In most cases injury of the hepatic artery (often the right hepatic artery) necessitates partial resection of the liver and frequently due to decreased blood supply to the central bile ducts also additional resection of the bifurcation of the common bile duct as well. In our cohort 18 of 20 patients had to undergo partial resection of the liver due to a concomitant vascular lesion, usually to the right hepatic artery. In these cases angio-CT showed a remarkable demarcation and intraoperative inspection a visibly impaired arterial perfusion of the right liver lobe. In one female patient partial resection of the liver was not performed because intrahepatic arterial perfusion was not significantly impaired as demonstrated by angio-CT results as well as intraoperative inspection. Apparently, sufficient collaterals ensured adequate arterial perfusion of the right liver lobe. Generally, the right hepatic artery supplies the bile ducts of the right liver lobe and segment IV. In our experience injury to the right hepatic artery virtually always results in secondary cholangitis with its consecutive complications. In our series the indication for partial resection of the liver is a result of questionable perfusion of the bile duct. In our view, arterial perfusion of the central bile ducts appears also to be a prerequisite for the healing process of biliodigestive anastomosis. In our view end-to-end reconstruction of the hepatic artery should only be considered in cases with fresh arterial lesions. This approach requires immediate detection of vascular injuries.

At least in our view none of the conventional classifications of iatrogenic bile duct lesions is able to differentiate the extent of iatrogenic bile duct lesions in sufficient detail to provide clear treatment guidelines based on comparative studies. This lack of relevant detail includes the lack of consideration for the variance of possible lesion combinations, including vascular injuries [3, 5, 9, 20, 21]. Until today the most frequently cited classification is the Strasberg classification, which is based mainly on the classification according to Bismuth [3]. The Bismuth classification was developed to describe the degree of bile duct lesions in terms of fixed strictures following open cholecystectomy [9]. The advantage of the Strasberg classification is the comprehensive demonstration of bile duct lesions. In our view, the subclassification of detailed lesions of aberrant right bile ducts is inappropriate as this anatomic variance is evident in only 2% of the normal population [3, 22]. For the reasons outlined above we consider the coincidence of concomitant vascular injuries with a frequency rate of 11–32% as much more relevant for proper therapeutic decision making as well as for prognostic considerations. The Strasberg classification does not consider additional vascular lesions. The Neuhaus classification also refrains from classifying concomitant vascular injuries, but characterises in more detail the extent and localisation of bile duct lesions as compared to the Strasberg classification. However, the Neuhaus classification also does not include the extent of lesions including the level of the lesion, for example, a lesion above the hepatic duct bifurcation [5]. In comparison, the classification developed by Siewert includes concomitant vascular injuries, but has weaknesses in the detailed description of bile duct lesions. Therefore, classification of some lesions, for instance of an anatomically aberrant right bile duct, is not always possible. Basically, the same applies to the Steward-Way classification [18, 20, 21].

To categorise our patients, we used the Hanover Classification, which we have developed and validated. Figures 1–5 illustrate this classification. This classification permits us to look at the complete extent of the lesion including possible additional vascular injuries. As this classification also comprises lesions above the bifurcation, we were able to categorise patients, who were not categorised in any of the existing classifications so far. In our collective 17 patients could be classified in this category (D4 lesions), for example, extensive lesions of the bile duct above the bifurcation as well as accidental resections of the bifurcation. Surgical treatment of this type of injury pattern is particularly demanding.

The majority of patients treated in our institution remained symptom-free during follow-up. But in further 19 cases mostly minor surgical interventions were required during the first hospitalisation in our institution. This correlates with data published by other large centres [6, 8, 19]. We consider therefore that our long-term results reflect the adequacy of the actually chosen definitive surgical treatments in our series.

As we have demonstrated here the extent of iatrogenic bile duct lesions is very variable. As this series shows, most iatrogenic bile duct lesions that are referred to a tertiary referral centre are usually very serious complications. The Hannover Classification may be helpful to standardize the systematic description of these lesions in order to establish evidence-based generalized lesion-type-specific treatment recommendations. It must be underlined that this current study is biased by the fact that it was performed at a referral centre for hepatobiliary surgery and may therefore not reflect how iatrogenic bile duct injuries are managed at the national level.

##  Conflict of Interests

Drs. H. Bektas, M. Kleine, A. Tamac, J. Klempnauer, and H. Schrem have no conflict of interests or financial ties to disclose.

## Figures and Tables

**Table 1:** Shown is the frequency of identification of the cystic duct (d. cysticus) and/or the cystic artery (a. cystica) prior to transsection during cholecystectomy which was followed by the diagnosis of an iatrogenic bile duct lesion. The frequencies of identification were determined in this study with the available operating reports.

Structure	Positively identified (*n*)	Not positively identified (*n*)	No comments (*n*)	Identification questionable (*n*)
D. cysticus	59	11	23	—
A. cystica	54	8	30	1

**Table 2:** Therapeutic methods and results of 93 patients with bile duct reconstruction after iatrogenic bile duct injuries.

Primary therapy in the local hospital	*n* = 93
Exclusive endoscopic therapy with stent	8
Explorative laparotomy +	29
*and transfer without reconstruction *	*10 *
*and suture *	*5 *
*and T-drain *	*9 *
*and drain *	*5*
Drainage only	3
Hemihepatectomy + biliodigestive anastomosis	1
E/E reconstruction	6
Biliodigestive anastomosis	18
No further primary therapy	28

Therapy after referral to our centre	*n* = 93

Explorative laparotomy and removal of a clip	2
Explorative laparotomy and suturing	5
Explorative laparotomy and adhesiolysis	3
Drainage only	1
Hepaticojejunostomy	53
Hepaticojejunostomy and reconstruction of a. hep. com	1
Right hemihepatectomy only	2
Liver segment resection	2
Hemihepatectomy with hepaticojejunostomy	13
Re-hepaticojejunostomy	9
Whipple's procedure	2

Subsequent interventions at our centre	*n* = 17

Re-hepaticojejunostomy	4
Partial resection of the liver	1
Liver transplantation	2
Incisional hernia	7
Closure of a tracheostoma	2
Relaparotomy due to adhesion ileus	1

Complications requiring revision	*n* = 19

Secondary haemorrhage	3
Bileleak	7
Anastomotic insufficiency of a hepaticojejunostomy	2
Peritonitis	4
Duodenal perforation	2
Obstruction of the hepatic artery and the portal vein	1

Hospital lethality	*n* = 3

Primarily injured common hepatic artery followed by sepsis in all cases	

Follow-up period	*n* = 63

Symptom-free	38
Symptoms due to adhesions	7
Pain in the region of the scar	3
Recurrent cholangitis	15

**Table 3:** Shown is a summary of specifically proposed initial surgical approaches for different types of bile duct lesions as classified by the Hanover classification versus the actually performed primary or secondary surgical treatment in our study.

Proposed initial treatment according to the Hanover Classification	Type of injury	*n* (93)	Actually performed primary or secondary treatment in our institution
	*Bile leakage*		

Drainage alone or direct suture of leak with or without *t*-tube placement →66 % congruence between theory and practice	A1	7	4x drainage alone or direct suture of leak with or without t-tube placement 3x hepaticojejunostomy at the level of the common hepatic duct
	A2	5	1x hepatic segmentectomy 4x drainage alone or direct suture of leak with or without t-tube placement

	*Stenosis of the common bile duct*		

Removal of clips, drainage, and stenting or T-tube drainage of the bile duct. In case of necrosis of the duct wall: resection and primary reconstruction or hepaticojejunostomy. →75 % congruence between theory and practice	B1	1	1 x resection of the bifurcation of the common bile duct and hepaticojejunostomy
	B2	3	2x resection of the bifurcation of the common bile duct and hepaticojejunostomy 1x right anatomical liver resection and resection of the bifurcation of the common bile duct and hepaticojejunostomy

	*Tangential injury*		

Primary reconstruction with drainage and stenting of the bile duct or hepatico-jejunostomy. In case of injury of the right hepatic artery a liver resection is usually necessary →90% congruence between theory and practice	C1	1	1x hepaticojejunostomy at the level of the common hepatic duct
	C2	5	3x hepaticojejunostomy at the level of the common hepatic duct 2x Whipple's procedure
	C2d	1	1 x hepaticojejunostomy at the level of the common hepatic duct
	C3	2	1x resection of the bifurcation of the common bile duct and hepaticojejunostomy 1x hepaticojejunostomy at the level of the common hepatic duct
	C4	3	1x resection of the bifurcation of the common bile duct and hepaticojejunostomy 2x hepaticojejunostomy at the level of the common hepatic duct

	*Complete transection of the common bile duct*		

Primary end to end reconstruction with stenting and drainage or hepaticojejunostomy. In case of injury of the right hepatic artery, a liver resection is usually necessary →72% congruence between theory and practice	D1	7	2x resection of the bifurcation of the common bile duct and hepaticojejunostomy 4x hepaticojejunostomy at the level of the common hepatic duct 1x right anatomical liver resection and resection of the bifurcation of the common bile duct and hepaticojejunostomy
	D2	19	1x resection of the bifurcation of the common bile duct and hepaticojejunostomy 18x hepaticojejunostomy at the level of the common hepatic duct
	D2d	6	2x hepaticojejunostomy at the level of the common hepatic duct 3x right anatomical liver resection and resection of the bifurcation of the common bile duct and hepaticojejunostomy 1x hepaticojejunostomy at the level of the common hepatic duct with additional arterial reconstruction of the right hepatic artery
	D3	7	2x right anatomical liver resection and resection of the bifurcation of the common bile duct and hepaticojejunostomy 4x right anatomical liver resection and resection of the bifurcation of the common bile duct and hepaticojejunostomy 1x hepaticojejunostomy at the level of the common hepatic duct
	D3d	2	1x hepaticojejunostomy at the level of the common hepatic duct 1x resection of the bifurcation of the common bile duct and hepaticojejunostomy with additional reconstruction of the right hepatic artery and the portal vein.
	D3 d + pv	1	1x right anatomical liver resection and resection of the bifurcation of the common bile duct and hepaticojejunostomy
	D3pv	1	1x resection of the bifurcation of the common bile duct and hepaticojejunostomy
	D4	9	2x hepaticojejunostomy at the level of the common hepatic duct 7x resection of the bifurcation of the common bile duct and hepaticojejunostomy
	D4c	1	1x hepatic segmentectomy
	D4d	6	4x right anatomical liver resection and resection of the bifurcation of the common bile duct and hepaticojejunostomy 2x resection of the bifurcation of the common bile duct and hepaticojejunostomy
	D4d + pv	2	2x right anatomical liver resection and resection of the bifurcation of the common bile duct and hepaticojejunostomy

	*Strictures of the common bile duct*		

Stenting or hepaticojejunostomy →100% congruence between theory and practice while initial therapy in primary hospitals	E1	0	
	E2	3	3x hepaticojejunostomy at the level of the common hepatic duct
	E3	1	1x resection of the bifurcation of the common bile duct and hepaticojejunostomy
	E4	0	
